# Human presence impacts fungal diversity of inflated lunar/Mars analog habitat

**DOI:** 10.1186/s40168-017-0280-8

**Published:** 2017-07-11

**Authors:** A. Blachowicz, T. Mayer, M. Bashir, T. R. Pieber, P. De León, K. Venkateswaran

**Affiliations:** 1 Biotechnology and Planetary Protection Group, Jet Propulsion Laboratory, California Institute of Technology, 4800 Oak Grove Dr., M/S 89-2, Pasadena, CA 91109 USA; 2 Department of Pharmacology and Pharmaceutical Sciences, School of Pharmacy, University of Southern California, Los Angeles, CA 90089 USA; 3 Division of Endocrinology and Metabolism, Medical University Graz, Graz, Austria; 4 Department of Space Studies, University of North Dakota, Grand Forks, ND 58202 USA

**Keywords:** Closed habitat, Surface, Mycobiome, Succession

## Abstract

**Background:**

An inflatable lunar/Mars analog habitat (ILMAH), simulated closed system isolated by HEPA filtration, mimics International Space Station (ISS) conditions and future human habitation on other planets except for the exchange of air between outdoor and indoor environments. The ILMAH was primarily commissioned to measure physiological, psychological, and immunological characteristics of human inhabiting in isolation, but it was also available for other studies such as examining its microbiological aspects. Characterizing and understanding possible changes and succession of fungal species is of high importance since fungi are not only hazardous to inhabitants but also deteriorate the habitats. Observing the mycobiome changes in the presence of human will enable developing appropriate countermeasures with reference to crew health in a future closed habitat.

**Results:**

Succession of fungi was characterized utilizing both traditional and state-of-the-art molecular techniques during the 30-day human occupation of the ILMAH. Surface samples were collected at various time points and locations to observe both the total and viable fungal populations of common environmental and opportunistic pathogenic species. To estimate the cultivable fungal population, potato dextrose agar plate counts method was utilized. The internal transcribed spacer region-based iTag Illumina sequencing was employed to measure the community structure and fluctuation of the mycobiome over time in various locations. Treatment of samples with propidium monoazide (PMA; a DNA intercalating dye for selective detection of viable microbial populations) had a significant effect on the microbial diversity compared to non-PMA-treated samples. Statistical analysis confirmed that viable fungal community structure changed (increase in diversity and decrease in fungal burden) over the occupation time. Samples collected at day 20 showed distinct fungal profiles from samples collected at any other time point (before or after). Viable fungal families like *Davidiellaceae*, *Teratosphaeriaceae*, *Pleosporales*, and *Pleosporaceae* were shown to increase during the occupation time.

**Conclusions:**

The results of this study revealed that the overall fungal diversity in the closed habitat changed during human presence; therefore, it is crucial to properly maintain a closed habitat to preserve it from deteriorating and keep it safe for its inhabitants. Differences in community profiles were observed when statistically treated, especially of the mycobiome of samples collected at day 20. On a genus level *Epiccocum*, *Alternaria*, *Pleosporales*, *Davidiella*, and *Cryptococcus* showed increased abundance over the occupation time.

**Electronic supplementary material:**

The online version of this article (doi:10.1186/s40168-017-0280-8) contains supplementary material, which is available to authorized users.

## Background

Planning future space explorations, involving potential human missions to Mars, would require constructing a safe closed habitat [1, 2]. An inflatable lunar/Mars analog habitat (ILMAH) is a unique, simulated closed environment (isolated by HEPA filtration) that can be utilized to overcome challenges associated with both technical and scientific issues [3]. Because the ILMAH mimics International Space Station (ISS) conditions and is treated as a prototype habitat for future space explorations, microbiological characteristics of such a closed environment is of high interest to the National Aeronautics and Space Administration (NASA). The environmentally controlled ILMAH is an easily accessible system that enables samples to be collected and analyzed at multiple times at relatively low cost. Understanding the microbiome of a closed system and its association with human inhabitation will help to assess the correlation between human health and microbiome of the habitat as well as the influence of microorganism on the habitat deterioration [4–6].

The highly specialized structure of the simulated ILMAH keeps its inhabitants in isolation from the outside environment. Except for the exchange of the air between outdoor and indoor environments via an advanced environmental control system, the ILMAH mimics the ISS and other future habitats of human explorers on the other planets [3]. This unique feature of the ILMAH allows observing the changes in the microbiome during human occupation. The bacteriome of the ILMAH was recently reported [7], as in the case of most of the studies reporting on bacterial microbiomes [8]. The molecular fungal diversity of Japanese Experimental Module—Kibo, on the ISS, revealed abundance of fungi associated with astronauts, but succession of viable fungal population in their habitat was not addressed [9]. The skin fungal microbiota of 10 Japanese astronauts showed temporal changes before, during, and after their stay on the ISS. The molecular fungal diversity associated with various body parts was reduced during the spaceflight when compared to pre-flight data. However, the ratio of *Malassezia* genetic signatures to all fungal gene copies (including dead fungal cells) increased during their stay at the ISS—but the viability of these fungi was not confirmed [10]. This is the first report that thoroughly characterizes the mycobiome of a simulated habitat meant for the future human habitats on other planets.

Utilization of next generation sequencing (NGS) techniques enables more in-depth analysis of indoor microbiomes [11]. Many studies focus on the bacterial microbiome of intensive care units [8, 12–14], pharmaceutical clean rooms [15–17], or tissue banks [18] since their microbial composition has an impact on human health and life. Nosocomial infections acquired in hospitals and other health care facilities remain the sixth leading cause of death in the hospitals in USA [19, 20]. Nosocomial infections are mostly caused by various fungal species that belong to the *Candida* genus and filamentous fungus *Aspergillus fumigatus* [21–23]. Therefore, it remains important to screen future closed habitats for the presence of opportunistic pathogens that can affect health of immunocompromised astronauts. So far, majority of the indoor microbiome studies have focused on the bacterial microbiome without analyzing the mycobiome. In addition, those few studies that characterized fungi of indoor environments focused on culture-based populations [24–27]. In those cases, where new molecular techniques were implemented [28–31] viable fungi were not differentiated from the total population (viable and dead) [32]. The internal transcribed spacer region-based iTag Illumina sequencing coupled with the propidium monoazide (PMA) treatment used in this study can determine the viable mycobiome.

Fungi are extremophiles that can survive harsh conditions such as low nutrient [33], desiccation [34], high/low temperatures [35, 36], acidic/alkaline [37, 38], radiation [39, 40], and other environments [41, 42]. Fungal species not only have been isolated from all known environments on Earth, including barren lands like deserts, caves, or nuclear accident sites, but also are known to be difficult to eradicate from other types of environments including indoor and closed spaces [8, 36, 42, 43].

Characterizing and understanding possible changes to, and succession of, fungal species in the ILMAH is of high importance since some of the fungi are extremophiles that are not only potentially hazardous to inhabitants but also can deteriorate the habitat itself [25, 44, 45]. It was previously reported that people spending a significant amount of time indoors might suffer from so called “sick building syndrome” (SBS). SBS is characterized by health- and comfort-related syndromes (e.g., headache, tiredness) that ease after leaving a building. Fatigue and discomfort might be caused not only by physical characteristics of the closed system (humidity, temperature, lighting) but also by biological contamination from both bacteria and fungi [46, 47]. Fungal pathogens’ presence in indoor areas might pose health hazards for people exhibiting immunodeficiency [48]. Pathogenic fungi produce a range of secondary metabolites (SMs) that influence their virulence (e.g., melanins, siderophores, or species-specific toxins), induce allergies, and cause diseases (e.g., aspergillosis, candidiasis, or cryptococcosis) [48, 49]. Prolonged stays in closed habitats (e.g., ILMAH, ISS, etc.) might be stressful for inhabitants and lead to a decrease in immune response; therefore, assessing the presence of any opportunistic pathogens is vital [50].

Previous reports on the mycobiome in NASA clean rooms and on the ISS documented NGS results from samples collected from various locations, but none of the studies focused on the analysis of the microbial succession of systematically collected samples [28, 32]. The bacterial and archaeal microbiome succession of the ILMAH using NGS has been carried out [7]. This is the first report characterizing the succession of fungi in a simulated closed system meant for human habitation on other planets utilizing both traditional and state-of-the-art molecular techniques. In addition, attempts were made during this study to elucidate the temporal and spatial distribution of the fungal population and diversity in a closed human habitat.

## Methods

### The ILMAH habitat

The physical characteristics of the ILMAH, along with detailed sampling procedures and periodicity, were previously described [7]. In brief, the ILMAH is located in Grand Forks, ND (47.9222 N, 97.0734 W) and has dimensions of 12 m by 10 m by 2.5 m. It contains a sleeping compartment, kitchen, toilet, and laboratory area. The ILMAH has its own ventilation system that pressurizes the habitat, provides breathing air for the crew, and removes unwanted material from the air stream. The ILMAH uses a blower that takes ambient air for pressurization and breathing air provision. It provides a positive pressure at 1 PSID (pressure differential) above normal atmosphere inside the habitat. The ambient air is pressurized by an industrial fan and sent to a standard HEPA flat panel particulate filter (Bryant GAPBBCAR2025, Honeywell, Morris Plains, NJ). This filter is replaced before each mission lasting up to 30 days. Maintenance personnel changes the filter from the outside. Since the humidity inside the habitat is not removed, water vapor during the months when the analog missions takes place range from 35 to 55%.

Three student crews inhabited the ILMAH for 30 days. During that period of time, there was no exchange between the interior and exterior environment except pumping the air filtered via ILMAH’s advanced controlling system [3]. In addition, students did not leave the ILMAH at any time for 30 days, and nothing came in or out, including food, people, water, or any supplies, except filtered air. Surface samples were collected consecutively during four sampling events (day 0 [T_0_], day 13 [T_13_], day 20 [T_20_], and day 30 [T_30_]) from eight designated sampling locations. Prior to inhabitation, the ILMAH surfaces were cleaned with 10% bleach and during the experiment period, it was cleaned weekly with antibacterial wipes.

### Sampling materials and procedure

Samples were collected using biological sampling kits (BiSKits, Quicksilver Analytics Inc., Abingdon, MD) previously documented as an efficient sampling device for surfaces [51]. All the BiSKits were prepared following the procedure described elsewhere [7, 51]. Briefly, the sterile phosphate buffer saline (PBS) provided by the distributor was discarded from the bottle and replaced with 15 mL of sterile UltraPure DNA free PBS (MoBio Laboratories Inc, Carlsbad, CA). Each BiSKit was rinsed once with PBS that was later collected into a sterile 15-mL falcon tube and kept at 4 °C as a background measure for biological materials associated with macrofoam (sampling device control). This precautionary step was required to overcome, if encountered, microbial contamination associated with sampling devices and other processing reagents. BiSKits prepared as described above were then packed into sterile zip lock bags and shipped at 4 °C to the University of North Dakota where they were kept at 4 °C till the experiment was carried out (within 2 to 3 days).

**Fig. 1:**
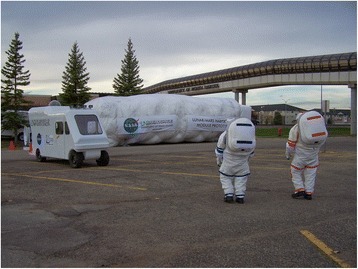
Picture of the closed habitat from outside

The ILMAH architecture was previously described [7] (see Additional file 1: Figure SF1, Fig. 1). Surface samples were collected from eight locations at four consecutive samplings: day 0 (prior to inhabitation), day 13, day 20, and day 30 during the inhabitation and right before ending the experiment. Originally, sampling activities were scheduled at regular intervals days 0, 10, 20, and 30 of human occupation; however, day 10 sampling scheduled on Thursday was delayed to day 13 (Sunday) to avoid risk related to shipping the samples over the weekend. The work schedule of the ILMAH crew members was regulated and is detailed in Additional file 2. Each location (surface area 1 m^2^) was sampled with one BiSKit in three directions following the same steps, horizontally from the left to the right, vertically from the bottom to the top, and diagonally from the right bottom corner to the left upper corner. After sampling, the sampled BiSKit device was extracted with sterile PBS and sampling fluids were collected in sterile 50-mL falcon tubes. Each BiSKit was washed and extracted twice with the sterile PBS giving approximately 45 mL of unconcentrated sample. Likewise, the BiSKit left open in the sampling area for the time necessary for sampling one location was treated as a field control; the unopened BiSKit was treated as a sampling device (BiSKit) control. Collected samples along with the controls were stored at 4 °C and sent overnight to JPL via cold shipping for further analysis. Sample processing for the microbiological analyses was started within 24 h from the sample collection.

### Sample processing

The extruded liquid samples from the BiSKit sampler (~45 mL per sample) were concentrated using an InnovaPrep Concentrating Pipette (Innova Prep LLC, Drexel, MO) to a final volume of ~4 mL. Appropriate aliquots of concentrated samples were further used for cultivation (200 μL) and molecular analyses (3 mL).

- Cultivable fungal burden and diversity
  For cultivation assay, samples were serially diluted by 10 and 100 times. One hundred microliters of each dilution was pour plated in duplicates on potato dextrose agar (PDA) and grown at room temperature (~25 °C). Colony-forming units (CFUs) were counted after 7 days of incubation, and cultivable fungal population was calculated per square meter of the sampling area. Simultaneously, up to 5 colonies exhibiting different morphologies were picked and stored as stab cultures in one-tenth semi-solid PDA medium. Cultivable isolates were identified using primers ITS 1F (5′-CTT GGT CAT TTA GAG GAA GTA A-3′) and Tw13 (5′-GGT CCG TGT TTC AAG ACG-3′) that target small and large subunit rRNA gene-coding regions of the small and large ribosomal subunit, including the internal transcribed spacers ITS1 and ITS2 [52, 53]. DNA was extracted using freezing (−80 °C) and thawing (+80 °C) cycle of fungal suspension in PBS for 15 min. This process was repeated 3 times. In some cases when DNA was not extracted by the freezing-thawing method, a PowerSoil® DNA Isolation Kit (MoBio) was used according to manufacturer’s instructions. PCR conditions were as follows: 95 °C for 3 min followed by 25 cycles of 95 °C for 30 s, 58 °C for 30 s, 72 °C for 2 min, and final elongation at 72 °C for 10 min. Amplified products were visualized by gel electrophoresis. PCR products were then enzymatically purified by using 40 IU of Exonuclease I (*E. coli* 20,000 IU/mL New England BioLabs, Inc. Ipswich, MA) and 8 IU of Antarctic Phosphatase (5,000 IU/mL, New England BioLabs, Inc.) per 20 μL amplification product. Heat reactions were carried out in a thermocycler as follows: 37 °C for 30 min, 80 °C for 15 min. Traditional Sanger sequencing was performed at Macrogen (Rockville, MD). The sequences were merged using DNAStar (Madison, WI), identified using UNITE fungal database [54], and aligned using ClustalW. A phylogenetic tree was constructed using MEGA6.06-mac applying neighbor-joining method [55]. Sequences from one representative of each strain and corresponding type strain were used to create the phylogenetic tree.
- DNA extraction
  The concentrated environmental samples (3 mL) were split into two equal parts. One half of the sample (1.5 mL) was treated with 12.5 μL of 2 mM PMA dye (Biotum, Inc., Hayward, CA), and the other half was left untreated. The final concentration of PMA in each treated sample was 25 μM. After PMA addition, both treated and untreated samples were kept in the dark for 5 min at room temperature and subsequently exposed to light in the PHaST Blue-Photo activation system for tubes (GenIUL, S.L, Terrassa, Spain) for 15 min. PMA-treated samples represent viable microorganisms whereas the non-PMA-treated samples represent the total number of viable and dead microorganisms [56]. After photo activation, each sample was split into two aliquots of 0.75 mL each. One aliquot of each sample was subjected to bead beating for 60 s at 5 m/s on the Fastprep-24 bead-beating instrument (MP Biomedicals, Santa Ana, CA). The solution after bead beating was combined with not bead-beated aliquots (1.5 mL), and then used for DNA extraction using the Maxwell-16 MDx automated system following manufacturer’s instructions (Promega, Madison, WI). Purified DNA was eluted into a final volume of 50 μL of Ultra Pure molecular water and divided into 4 aliquots that were stored at −80 °C.
- Molecular fungal community analysis using Illumina sequencing
  To determine fungal populations, a two-step amplification process was applied prior to MiSeq Illumina sequencing at Research and Testing Laboratory (RTL, Lubbock, TX). The forward primer was constructed with the Illumina i5 sequencing primer (5′-TCG TCG GCA GCG TCA GAT GTG TAT AAG AGA CAG-3′) and the ITS1F primer (5′-CTT GGT CAT TTA GAG GAA GTA A-3′) [57]. The reverse primer was constructed with the Illumina i7 sequencing primer (5′-GTC TCG TGG GCT CGG AGA TGT GTA TAA GAG ACA G-3′) and the ITS2aR primer (5′-GCT GCG TTC TTC ATC GAT GC-3′) [58]. Amplifications were performed in 25 μL reactions with Qiagen HotStar Taq master mix (Qiagen Inc, Valencia, CA), 1 μL of each 5 μM primer, and 1 μL of template. Reactions were performed on ABI Veriti thermocyclers (Applied Biosytems, Carlsbad, CA) under the following thermal profile: 95 °C for 5 min, then 25 cycles of 94 °C for 30 s, 54 °C for 40 s, 72 °C for 1 min, followed by one cycle of 72 °C for 10 min and 4 °C hold.
  Products from the first stage amplification were added to a second PCR based on qualitatively determined concentrations. Primers for the second PCR were designed based on the Illumina Nextera PCR primers as follows: Forward –AAT GAT ACG GCG ACC ACC GAG ATC TAC AC [i5index] TCG TCG GCA GCG TC and Reverse – CAA GCA GAA GAC GGC ATA CGA GAT [i7index] GTC TCG TGG GCT CGG. The second stage amplification was run the same as the first stage except for 10 cycles.
  Amplification products were visualized with eGels (Life Technologies, Grand Island, NY). Products were then pooled equimolar, and each pool was size selected in two rounds using Agencourt AMPure XP (BeckmanCoulter, Indianapolis, IN) in a 0.7 ratio for both rounds. Size-selected pools were then quantified using the Quibit 2.0 fluorometer (Life Technologies) and loaded on an Illumina MiSeq (Illumina, Inc. San Diego, CA) 2 × 300 flow cell at 10 pM.
- Bioinformatic and statistical analysis of fungal cultivable counts and Illumina sequences
  To assess the difference between fungal abundances in cultivable sample categories (based on time and location), the following univariate statistical analyses were carried out (https://www.r-project.org/). The normal distribution of the populations was tested using the Shapiro-Wilk normality test, and as most were not normally distributed (*p* value <0.05), we used a Kruskal-Wallis test coupled to a Dunn’s test (https://cran.r-project.org/web/packages/dunn.test/index.html) to investigate differences in the tested populations. Resulting *p* values were corrected using the Benjamini-Hochberg correction [59].
  A total of 8,426,774 raw paired reads were processed with mothur v.1.36.1 [60]. The 250 bp paired reads were merged by aligning the reads and correcting discordant base calls by requiring one of the base calls to have a Phred quality score at least 6 points higher than the other. Sequences shorter than 200 bp, having more than 8 homopolymers or containing ambiguous base pairs were excluded from the dataset. Subsequently, reads were pre-clustered [61] by combining low-abundant sequences that differed by 3 or less bases of a more abundant sequence. Chimeric sequences in each sample were identified by UCHIME [62] and also excluded from the dataset. Reads were classified using the ribosomal database project (RDP) classifier II [63] implementation of mothur and UNITE fungal rDNA database [54]. Non-fungal sequences were subsequently removed. ITSx was used to exclude non-ITS sequences from the dataset prior to clustering unaligned sequences into operational taxonomic units (OTUs) with a distance of 0.03 [64] using mother v.1.36.1. The nearest neighbor (single-linkage) algorithm was used for this. OTUs were classified by using the consensus taxonomy of all sequences assigned [60].
  An in-house R-script employing the libraries vegan, ape, gplots, mgcv, and GUniFrac was used to compare the fungal Illumina data (Additional file 3) [65, 66]. Each dataset consisting of the OTU abundances per sample was rarefied 1000 times to the lowest number of reads, and an average Bray-Curtis distance was calculated. This distance was then utilized to calculate nonmetric multidimensional scaling (NMDS) or principal coordinate analysis (PCoA), PERMANOVA (Adonis test), and multi-response permutation procedure (MRPP). In addition, the OTU abundances per sample of each dataset were sum normalized and used to employ either an analysis of variance (ANOVA) or a Spearman rank correlation on the statistically significant changing parameters and to generate a heat map (*p* value 0.05). The change of diversity was measured via the Shannon-Wiener diversity index. OTUs that were unclassified at phylum level were removed. Heat maps were presented at family level.

## Results

### Cultivable fungal burden and diversity

**Fig. 2:**
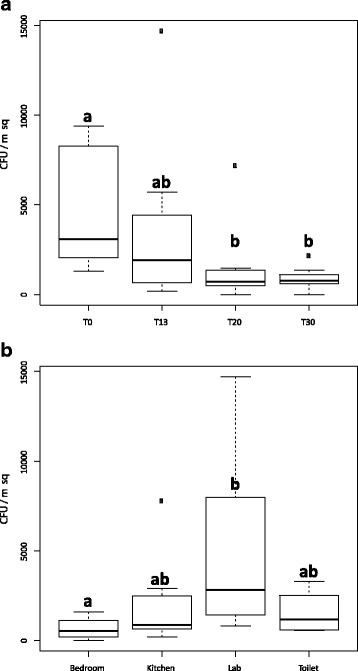
Statistical analysis of cultivable fungal diversity detected through the 30-day habitation period at all the locations based on colony-forming unit (CFU) counts. To assess the difference between fungal abundances in cultivable sample categories (based on time—**A** and location—**B**), we applied the following univariate statistics. The normal distribution of the populations were tested using Shapiro-Wilk normality test, and as most of them were not normally distributed (*p* value <0.05), we used a Kruskal-Wallis test coupled to a Dunn’s test to investigate differences in the tested populations. Resulting *p* values were corrected using the Benjamini-Hochberg correction. **A** CFU counts before crew occupation T_0_—*a* were statistically different from CFU counts at T_20_ and T_30_—*b*, but no statistical difference was observed between T_0_ and T_13_ counts—*ab*. Additionally, no statistical differences were observed between any other time points. **B** CFU counts in the bedroom—*a*, differed significantly from the CFU counts in lab—*b*, but no statistical differences were observed between bedroom and kitchen or toilet—*ab*. No statistical differences were observed between any other locations

The culture-based fungal abundance of the ILMAH was estimated for each sampling location with colony-forming unit (CFU) values ranging from below detection limit (BDL) to 10^4^ CFU/m^2^ (Additional file 4: Table ST1a). The highest abundance of the cultivable fungi was observed during the first sampling (T_0_) followed by ~1 log decrease in CFU during consecutive sampling events (T_13_, T_20_, T_30_) for each living compartment. High fungal population was noticed in samples collected at T_0_ (Fig. 2A). When statistically treated, the change in fungal abundance within different time points was significant (T_0_–T_20_ *p* = 0.008 and T_0_–T_30_ *p* = 0.0125) (Additional file 5: Table ST2a). The cultivable fungal population for the lab area was higher than observed in other compartments (Fig. 2B, Additional file 4: Table ST1b). A statistically significant difference was observed between the lab area and the bedroom (*p* = 0.0021) (Additional file 5: Table ST2b).

**Fig. 3:**
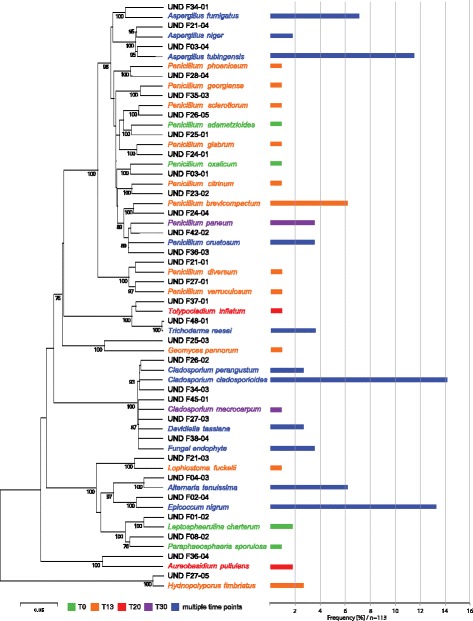
Cultivable fungal diversity detected through the 30-day habitation period at all the locations based on internal transcribed spacer (ITS) sequences. The phylogenetic tree was constructed using neighbor-joining method (bootstrap 1000). In total, 117 isolates were collected 113 of which were successfully sequenced (4 strains either did not show growth or did not respond to the sequencing methods attempted). The numbering of the isolates is explained as follows: F = fungi, first number (0–4) will be the sample collection day (0 = T_0_, 2 = T_13_, 3 = T_20_, 4 = T_30_), second number (1–8) will be sampling location, and the third number (1–5) is the replicate number of the isolate. For example, F23-02 will be a fungal strain, isolated from T_20_, at location number 3 and a second isolate. Frequency of isolates is given as a frequency bar after the name of fungus. *Colors of the bars* correspond to the collection time (single or multiple)

One hundred seventeen cultivable isolates were collected and identified targeting the ITS region. Screening sequences against the UNITE fungal database enabled identification of 32 species (Fig. 3). Among the cultivable isolates, only five strains had similarity lower than 97% that did not allow identification to the species level. All of the identified species but one—*Hydnopolyporus fimbriatus*—belong to the *Ascomycota* division (Fig. 3). The most abundant cultivable species were *Cladosporium cladosporioides* (16), *Epicoccum nigrum* (15), *Aspergillus tubingensis* (13), *Aspergillus fumigatus* (8), *Alternaria tenuissima* (7), and *Pennicillium brevicompactum* (7). *P. brevicompactum* was the only, out of the most commonly identified species, that was not present at multiple time points but only at T_13_ sampling (Fig. 3). Abundance of *C. cladosporioides* colonies increased during the ILMAH occupation whereas the abundance of *E. nigrum, A. tubingensis*, and *A. tenuissima* decreased. Another commonly isolated species, *A. fumigatus*, was not isolated during the last sampling event. Neither field nor sampling device controls showed cultivable isolates.

### Viable and total mycobiome (iTag Illumina-based analysis)

**Fig. 4:**
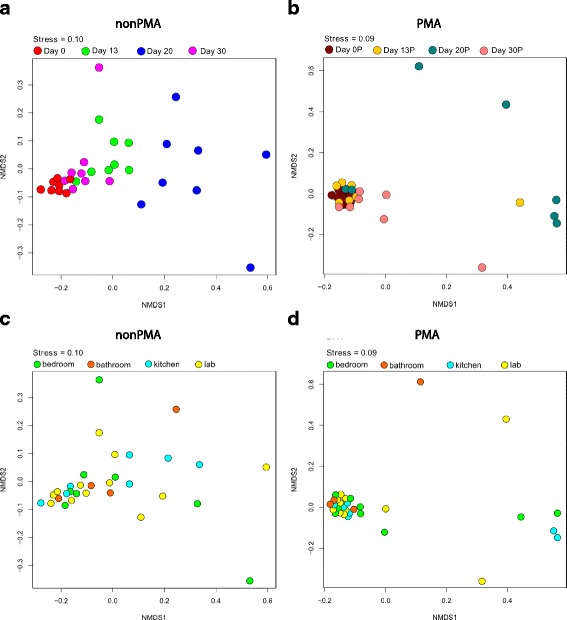
NMDS ordinations based on Bray-Curtis distances between all samples. **a** Ordination displaying the distance between non-PMA-treated samples taken at the different time points. **b** The distance between PMA-treated samples taken at the different time points. **c** The distance between non-PMA-treated samples taken at the different locations. **d** NMDS ordination displaying the distance between PMA-treated samples taken at the different locations. A “*P*” after the respective variable indicates that these are the samples treated with PMA. Plots **a**, **c**, and **b**, **d** represent the same data but differ in colors to underscore the focus on distribution over time and location, respectively

**Fig. 5:**
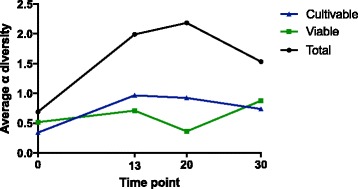
Linear representation of alpha diversity averages change over time for cultivable, viable and total mycobiome

The fungal richness of PMA-treated (viable) samples decreased when compared to untreated samples (dead and alive). In PMA-untreated samples, 98 families were detected, whereas OTUs belonging to 41 of these families were not viable. Moreover, both PMA-treated and PMA-untreated samples differed significantly in community relationships (NMDS analysis in Fig. 4, Adonis *p* value = 0.006 and MRPP, significance of delta = 0.002; *A* = 0.0419) and their Shannon diversity index indicated a significant reduction (paired *T* test *p* = 0.0000012) in viable fungal diversity. Observed differences in the PMA-treated (Fig. 4a, c) and PMA-untreated samples (Fig. 4b, d) indicate that untreated samples are overestimating the observed fungi. This observation was further confirmed when alpha diversity of cultivable, viable, and total mycobiome was plotted over time (Fig. 5). In this research communication, only viable mycobiome was considered and discussed in detail. Illumina-based reads of sampling device and field controls showed negligible signal from DNA contamination and hence not included in the following analysis.

### Viable fungal community structure

**Fig. 6:**
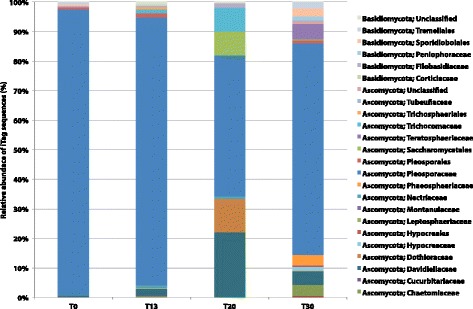
Dominant fungal population and succession patterns observed in 30-day occupation period of the ILMAH system. The OTUs presented in the bar graph are the most abundant. T_20_ surface samples show different fungal profile when compared to other time points. T_30_ samples show increase in fungal diversity when compared to other time point

The most abundant phylum that dominated the viable mycobiome of the ILMAH was *Ascomycota* (90% of all characterized OTUs) followed by *Basidiomycota* and unclassified fungi (~4 and ~5%, respectively). Incidence of the fungal OTUs at the family level for various time points and locations are presented in Table 1 and Table 2, respectively. The dominant *Pleosporaceae* (75% of all OTUs) along with unclassified fungi (5%) and *Davidiellaceae* (4%) constituted 84% of all OTUs present in PMA-treated samples (Tables 1 and 2 and Fig. 6). A closer look into the genus level of *Pleosporaceae* family indicated the domination of *Epicoccum* (92.95% of all OTUs) and *Alternaria* (6.8%) sequences. The dominant fungal OTUs in the ILMAH biome correspond with the most frequently isolated cultivable fungi (Figs. 3 and 6).

**Table 1:** Incidence of the fungal OTUs at the family level for various time points

Fungal phylum	Fungal family	Number of sequences							
		T_0_		T_13_		T_20_		T_30_	
		Total	Viable	Total	Viable	Total	Viable	Total	Viable
Ascomycota	Botryosphaeriales	115	31	1620				176	96
	Capnodiales		2	185	121			61	4353
	Chaetomiaceae			2415	1089	5533		1505	32981
	Cucurbitariaceae	451	221	2298	750	1879		2272	1256
	Davidiellaceae	2840	1423	42496	6099	129620	28495	55079	38510
	Dothioraceae	32	104	510	289	12864	14785	4726	688
	Hypocreaceae	174	68	2357	197	5510		3897	10952
	Hypocreales	73	86	9247	402	8566	1	7989	5638
	Leptosphaeriaceae	16	23	564	215	518	1	1639	1
	Lophiostomataceae	56	43	56		1850	1	26	
	Microascaceae			326		27007	9	735	6
	Montanulaceae	627	232	11273	621	22582		12697	77
	Nectriaceae	82	121	2890	1287	8479	804	2242	49
	Phaeosphaeriaceae	343	222	3551	105	6082	1	6969	30695
	Pleosporaceae	295115	380533	273232	251840	172919	61791	648825	619794
	Pleosporales	5202	3071	6876	4475	10056	13	12085	8859
	Saccharomycetales	17	33	346	3	45461	10414	17840	2092
	Sclerotiniaceae	52	16	355		1920		99	
	Teratosphaeriaceae	35	28	805	1	2	2	583	44749
	Trichocomaceae	68	32	10496	3461	14625	10495	12147	518
	Trichosphaeriales	3		122	1750			688	
	Unclassified	712	686	5082	640	4161		4450	6317
Basidiomycota	Filobasidiaceae	1164	419	5997	8	11570	1925	2550	4425
	Peniophoraceae	1							10916
	Polyporales	1				1339			
	Sporidiobolales	2563	1236	6556	315	22900	2	35692	24352
	Tremellales	7916	4158	42068	1030	72870	9	34932	18439
	Unclassified	409	272	2151	2171	481	504	2445	279
Unclassified	Unclassified	2973	10475	38015	17311	30832	10320	41608	53950

**Table 2:** Incidence of the fungal OTUs at the family level for locations

Fungal phylum	Fungal family	Number of sequences							
		Bedroom		Kitchen		Bathroom		Lab	
		Total	Viable	Total	Viable	Total	Viable	Total	Viable
Ascomycota	Capnodiales	215		26	121			5	4355
	Chaetomiaceae	171	32858	1668		2073		5541	1212
	Cucurbitariaceae	1186	16	2782	739	1248	57	1545	1415
	Davidiellaceae	71145	17039	40120	35104	53460	496	58969	21888
	Dothioraceae	4821	5774	161	286	172	9708	12970	98
	Herpotrichiellaceae	68	4	93	4	134	7	1407	28
	Hypocreaceae	979	11	4622	93	2095	10909	4133	204
	Hypocreales	9471	5617	8792	408	1712	12	5169	90
	Lophiostomataceae	30	21	71	19	9		1878	4
	Microascaceae	3086	3	6642	3	18317	3	23	6
	Montanulaceae	9231	116	5112	520	24292	68	7141	226
	Nectriaceae	216	1374	5618	3	1542	18	6177	866
	Phaeosphaeriaceae	1360	131	4209	55	6150	18	4318	30819
	Pleosporaceae	223517	357893	195638	299314	247789	199402	626137	457349
	Pleosporales	5933	4284	6768	590	7339	644	10668	10900
	Saccharomycetales	1904	1658	18978	10681	10035	49	29496	154
	Teratosphaeriaceae	94		55	9	13	8	932	44763
	Trichocomaceae	5453	1	11611	1167	8735	2236	10860	11102
	Others (20)	1835	5	494	12	530	16	365	26
	Unclassified	2007	426	5203	468	668	3105	5831	3644
Basidiomycota	Filobasidiaceae	3624	2198	935	120	8171	4412	8259	47
	Peniophoraceae			45	9922	79		1	994
	Polyporales	1						1339	
	Sporidiobolales	33860	24869	15193	173	10398	299	7923	564
	Tremellales	47729	17080	26291	2479	9002	1165	69144	2912
	Unclassified	1999	34	525	2172	1144	67	1459	953
Unclassified	Unclassified	37959	13825	17047	26040	22401	6260	29713	45931

Differences in the fungal community between samples were analyzed by multivariate statistics using ordination analyses and Monte Carlo-based permutation tests. Viable fungal communities showed similar mycobiome profiles throughout the sampling period except from the samples collected at T_20_, which were distinct (Fig. 4; Adonis *p* value = 0.001 and MRPP, significance of delta = 0.001 and *A* = 0.1168, Additional file 6: Figure SF2 MRPP: chance corrected within-group agreement A: 0.1626, significance of delta 0.001; Adonis: delta = 0.019). Interestingly, community profiles were similar between samples collected before crew occupation (T_0_) and at T_13_ and T_30_ (Additional file 7: Figure SF3; chance corrected within-group agreement A: 0.01802, significance of delta: 0.219, Adonis 0.228, Additional file 8: Table ST3).

**Fig. 7:**
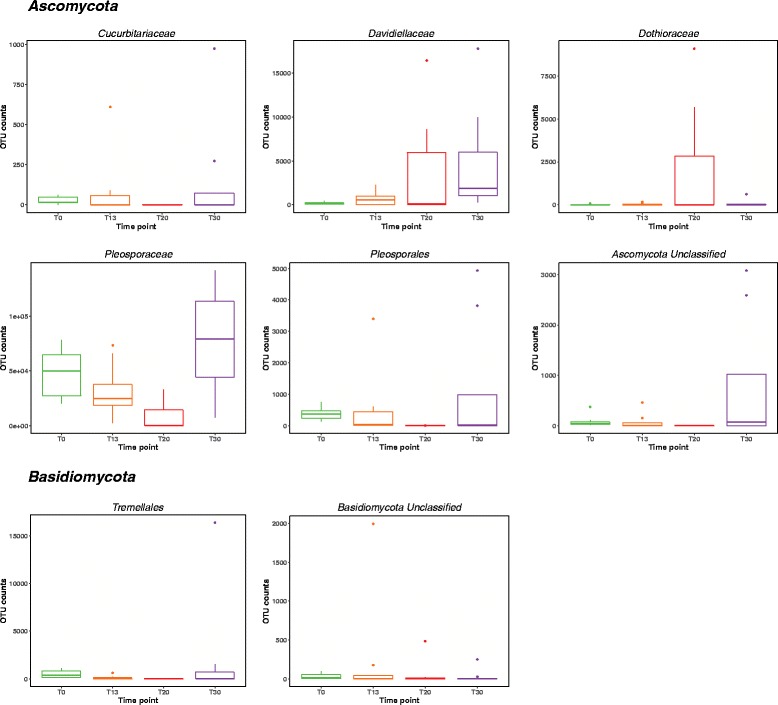
Box plots of viable dominant fungal families and their succession patterns observed in 30-day occupation period of the ILMAH system. The OTU counts presented in the boxplots are the most abundant. Each time point is represented in a different color: T_0_—*green*, T_13_—*orange*, T2_0_—*red*, T3_0_—*purple*

Observed differences in multivariate statistics led to the investigation of mycobiome changes on a single-OTU level. First, an Anova test carried out on samples that did not cluster with the rest (Fig. 4) showed presence of representatives of *Pleosporaceae*, *Pleosporales*, *Saccharomycetales*, *Tremellales*, and *Trichocomaceae* families. Second, throughout the inhabitation, the level of *Pleosporaceae* showed significant fluctuation—from 96 to 47% and 70% at T_0_, T_20_, and T_30_, respectively (Fig. 7). Additionally, while *Pleosporaceae* presence decreased to 47%, a significant increase in the levels of *Davidiellaceae* (22%), *Dothioraceae* (11%), *Saccharomycetales* (8%), and *Trichocomaceae* (8%) was observed when compared to other time points (Fig. 6, Additional file 9: Table ST4). Interestingly, the presence of less abundant families observed before crew occupancy (T_0_) increased at (T_30_), i.e., *Davidiellaceae* (4.45%), *Hypocreaceae* (1.26%), *Phaeosphaeriaceae* (3.54%), *Teratosphaeriaceae* (5.17%), and *Sporidiobolales* (2.81%), as well as members of new fungal families were observed, i.e., *Capnodiales* (0.5%), *Chaetomiaceae* (3.81%), and *Peniophoraceae* (1.26%) (Table 1 and Fig. 6, Additional file 9: Table ST4).

**Fig. 8:**
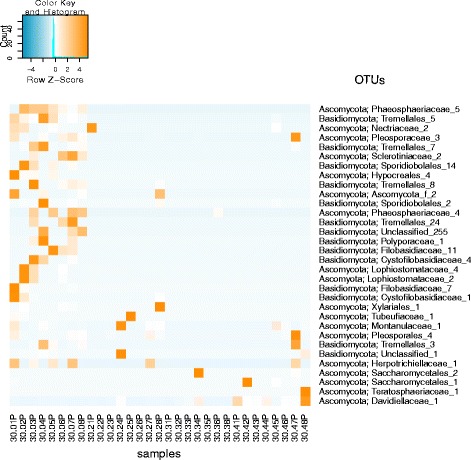
Heat map of the taxa that showed a significant correlation (*p* value 0.01) with the factor time in the PMA-treated sample set. The color *blue* indicates a low abundance of the single OTU in the respective sample, and *orange* indicates a high abundance of the single OTU in the respective sample. Each column represents one sample collected throughout the study. The numbering pattern is explained as follows 30 means 30-day study. The first number (0–4) will be the sample collection day (0 = T_0_, 2 = T_13_, 3 = T_20_, 4 = T_30_), second number (1–8) will be sampling location, and *P* stands for PMA-treated samples. For example, 30.06P will be a sample collected during the 30-day study from T_0_ at location 6

Spearman rank correlation was applied to each OTU’s abundance pattern and sampling event to determine significant correlation of viable fungal families with various time points. The results presented as a heat map contain 13 OTUs that showed a significant correlation with a *p* value of 0.01 and 32 OTUs with a *p* value of 0.05. All OTUs but *Davidiellaceae*, *Teratosphaeriaceae*, *Tremellales_3*, *Pleosporales*, and *Pleosporaceae* were more abundant during the sampling before the crew inhabited the ILMAH (Fig. 8).

Further investigation of OTU abundances on the genus level revealed changes in OTU counts over the course of time. The numbers of OTUs identified as *Epiccocum*, *Alternaria*, *Pleosporales*, and *Cryptococcus* fluctuated from high abundance at T_0_ to significantly lower counts at T_20_ and then counts increased again at T_30_. OTUs identified as *Davidiella* increased throughout the occupation time whereas OTU counts for *Aspergillus*, *Aureobasidium*, and *Candida* were increasing over time (peak at T_20_) with a drastic decrease at T_30_. Location wise before the ILMAH occupation, all compartments exhibited high abundance of *Epiccocum* OTUs that decreased over time and again increased at T_30_. *Alternaria* OTUs were less abundant than the ones identified as *Epiccocum* genus. Nevertheless, they showed the same fluctuation pattern in all compartments but bedroom. While accumulation of *Davidiella* OTU counts was observed in bedroom and lab, the OTU counts in the bathroom did not differ between the T_0_ and T_30_. At the same time, accumulation of *Davidiella* OTUs was observed in the kitchen with the highest abundance during the T_20_ sampling event.

To sum up, statistical analysis revealed differences in community structure between time points in particular between T_20_ and any other previous or following time point. *Davidiellaceae*, *Teratosphaeriaceae*, *Tremellales_3*, *Pleosporales*, and *Pleosporaceae* families were shown to increase over the occupation time period. On a genus level, *Epiccocum*, *Alternaria*, *Pleosporales*, *Davidiella*, and *Cryptococcus* showed increased abundance.

## Discussion

Understanding microbial characteristic of a controlled habitat, like the ILMAH, may facilitate discerning the microbial population dynamics as well as the development of appropriate countermeasures. Knowledge about the viable mycobiome will not only allow the development of required maintenance and cleaning procedures in the closed habitat but also prevent it from deteriorating and being a potential health hazard for its inhabitants [67]. Multiple studies have shown that use of propidium monoazide (PMA), a dye that can penetrate the compromised cell walls, enables more accurate analysis of the viable microbiome [68, 69]. It was also shown that PMA treatment might be successfully applied to determine dead and viable counts for various fungal species [70, 71]. As in this study, other report proved that DNA from dead cells, when not removed from samples, before molecular analyses, might overshadow the actual diversity since presence of less abundant microbial species were masked [32]. PMA-treated and PMA-untreated samples varied significantly in the ILMAH fungal community structures (*p* value = 0.006), and it had been reported before that low abundant species were detected in PMA-treated samples for bacteria, fungi, and viruses [66]. This approach validated the importance of PMA treatment to accurately determine the viable mycobiome of environmental samples. As a result, this study discussed only viable fungal communities to determine succession patterns and community structure over the course of time.

Various studies showed the positive correlation between changes in the indoor bacteriome and human presence [7, 28, 30, 66, 72] whereas such observation was not confirmed for the mycobiome [29, 30, 73]. Major sources of indoor microbiome were reported to be associated with human skin commensals transmitted via shoes, clothes, coughing, and talking [74, 75]. It was also shown that the indoor mycobiome was mostly influenced by airborne fungi rather than human presence and their shedding, despite the fact that fungi were associated with human skin, lungs, urogenital tract, oral nasal cavities, and the gut [30, 76–79]. In addition, few studies demonstrated that key determinants for mycobiome indoors might be the age of the built-in environments and the relative humidity, which can enhance fungal growth [80]. A recent 6-month survey on Japanese astronauts on the ISS revealed that the most abundant (dead or alive) fungal genus was *Malassezia* [10] whereas in this study, the most frequent viable fungal genera of closed habitat were *Epiccocum*, *Alternaria*, and *Pleosporales*, which are environmental organisms rather than human commensals.

The presence of fungi inside the closed habitat, houses, or any types of man-made buildings was correlated with the amount of the water (relative humidity; RH) present in the environment [80]. The environmental relative moldiness index (ERMI) increases with elevated amounts of water [81]. The most abundant genera in common households were *Alternaria*, *Cladosporium*, and *Epicoccum* whereas *Aspergillus* and *Penicillium* were predominant in water-damaged building [81]. Similarly, in this study (measured RH 32 to 55%), the most abundant fungal genera were *Epiccocum*, *Alternaria*, *Pleosporales*, and *Cryptococcus*, which could be compared with the previous observations for common households. Nevertheless, genera present in houses and closed habitats could have an impact on human health. These molds were associated with allergies and asthma [67, 82]. Elevated level of fungal allergenic molecules, such as enzymes, toxins, cell-wall components, and cross-reactive proteins could induce type I hypersensitivity [67]. Additionally, because of their ability to colonize the human body and produce toxins, volatile organic compounds, and proteases, the common molds (*Alternaria*, *Cladosporium*, *Epicoccum*) could damage airways of immunocompromised occupants, which make them more dangerous than any other allergenic source [82–85]. In this study, accumulation of *Alternaria* and *Epicoccum* genera over time was observed, which, in combination with reported decreased immunity in occupants of confined spaces, e.g., astronauts [50, 86] could lead to developing allergy and asthma symptoms.

Throughout this study, the abundance of dominant *Pleosporaceae* family members decreased (94% at T_0_ to 71% at T_30_), while other fungal families were observed to increase, possibly as a result of human presence. However, positive correlation between increased fungal diversity and human presence requires studying the mycobiome of the occupants. It might be possible that the implemented cleaning procedures including weekly dusting, sweeping, wet mopping the floor, and antibacterial wipes resulted in suppressed growth of *Pleosporaceae* members over the time. The community structure observed at T_20_ was distinct from other time points. Detailed logging data collected during 30-day mission did not show any abnormal accidents or cleaning activities preceding T_20_ sampling. The recorded data indicated that cleaning was conducted between the sampling events (~4–5 days prior to sampling). However, the inhabiting crew might have inadvertently cleaned just prior to T_20_ sampling and that might have removed both total and viable fungal populations (Table 1). The significant increase of *Davidiellaceae* (22%), *Dothioraceae* (11%), *Saccharomycetales* (8%), and *Trichocomaceae* (8%) sequences at T_20_ might be due to the fact that under-represented members became available for PCR reaction among the competing dominant fungal DNA.

Both *Cladosporium* sp. members of *Davidiellaceae* family and *Aurobasidium* sp. of *Dothioraceae* family have the capacity to survive extreme environments like the ice of the Antarctica [87] or radioactive explosion site of Chernobyl Power Plant accident [88]. Additionally, *Cladosporium* sp. and *Aureobasidium pullulans* were isolated from hypersaline waters with NaCl concentration reaching 25% indicating high osmotolerance [89, 90]. *Penicillium* sp. of *Trichocomaceae* family isolated from high-altitude soil in Indian Himalaya has been shown to tolerate a wide range of pH from 2 to 14 and a salt concentration between 10 and 20% [91]. Most of the *Apergillus* sp. of *Trichocomaceae* family is soil fungi or saprophytes [92], but there has been a recent report of isolation of *A. fumigatus* from ISS [32]. In-depth analysis of isolate ISSFT-021 and IF1SW-F4 revealed increased UV resistance (in preparation) and virulence in neutrophil-deficient larval zebrafish model of invasive aspergillosis [93]. All in all, the capacity to survive in such extreme environments might help the observed in the study *Davidiellaceae*, *Dothioraceae*, and *Trichocomaceae* species to adjust and survive hostile conditions of the ILMAH.

## Conclusions

Accumulation of viable mycobiome during the experiment duration and differences in fungal community profiles were observed. On a genus level, *Epiccocum, Alternaria, Pleosporales, Davidiella*, and *Cryptococcus* showed increased abundance over the occupation time. Unlike results of molecular analyses, cultivable fungi counts decreased over time. *Epicoccum nigrum* was the most dominant cultivable isolate; however, most of the fungal species detected via molecular approach were not cultured. During human presence, the overall fungal diversity has changed, that in the long run, may lead to a conclusion that proper maintenance protocols of a closed habitat may be required to preserve it from deteriorating and keep it safe for its inhabitants.

## Additional files

Additional file 1: Figure SF1. Schematic representation of the ILMAH architecture with dimensions. The sampling locations are indicated with stars and numbers (1, 2: Bedroom; 3, 4: Kitchen; 5: Bathroom; and 6, 7, 8: Laboratory). (PDF 911 kb)

Additional file 2: Crew schedule assignments for the 30 days. (PDF 911 kb)

Additional file 3: R code and script used as published in Weinmaier and Probst et al., 2015 Microbiome. (PDF 300 kb)

Additional file 4: Table ST1. Cultivable fungal characteristics of ILMAH surface samples. Tables represent: a) CFU counts for each sampling area during consecutive sampling events (before crew occupation, Day 13, Day 20 and Day 30). Sampling areas are numbered 1-8. 1 and 2 correspond to bedroom area, 3, 4 – kitchen, 5 – bathroom and 6-8 lab. CFU counts are reported per meter square; b) CFU counts for specific ILMAH compartments. Table contains average CFU counts for each compartment: bedroom, kitchen, bathroom and lab area (for example CFU counts from area 1 and 2 at Day 13 were used to calculate the average CFU count for the bedroom compartment at Day 13). CFU counts are reported per meter square. (PDF 52 kb)

Additional file 5: Table ST2. Statistical analysis to compare cultivable fungal populations of the different (a) time points and (b) locations. (a) CFU counts of cultivable fungal populations observed at various time point were compared to each other to asses if there are any statistically significant changes in CFU counts over the course of time; (b) CFU counts of cultivable fungal populations observed at different compartments were compared to each other to asses if there are any statistically significant changes in CFU counts between locations. (PDF 43 kb)

Additional file 6: Figure SF2. NMDS ordinations based on Bray-Curtis distances between non-PMA- and PMA-treated samples taken at different time points. The analysis shows a significant difference between the PMA treated and not treated samples and between the different time points but not between the different locations. A “P” after the respective variable indicates that these are the samples treated with PMA. (PDF 44 kb)

Additional file 7: NMDS ordinations based on Bray-Curtis distances between PMA treated samples without T_20_ taken at different time points. A “P” after the respective variable indicates that these are the samples treated with PMA. (PDF 30 kb)

Additional file 8: Statistical analysis of viable (PMA treated) samples to compare fungal populations of the different a) time points and b) locations. (a) Community profiles of viable fungal populations observed at various time point were compared to each other to asses if there are any statistically significant changes over the course of time; (b) Community profiles of viable fungal populations observed at different compartments were compared to each other to asses if there are any statistically significant changes between locations. Results marked with * are statistically significant. (PDF 44 kb)

Additional file 9: Table ST4. Percent change of OTU counts at family level. Information about the percent changes of the OTU counts of selected families during consecutive time points is presented. (PDF 45 kb)

Additional file 10: SRA numbers for individual samples. This file contains detailed information about accession number of each sample deposited within the study SPR069729. (TXT 12 kb)

Additional file 11: OTU deduced from the PMA treated samples. This file contains OTU counts of all PMA treated samples collected during the study. The numbering pattern is explained as follows 30 means 30-day study. The first number (0–4) will be the sample collection day (0 = T0, 2=T13, 3=T20, 4=T30), second number (1–8) will be sampling location, and P stands for PMA-treated samples. For example, 30.06P will be a sample collected during the 30-day study from T0 at location 6. (CSV 334 kb)

Additional file 12: OTU deduced from the PMA untreated samples. This file contains OTU counts of all samples not treated with PMA collected during the study. The numbering pattern is explained as follows 30 means 30-day study. The first number (0–4) will be the sample collection day (0 = T0, 2=T13, 3=T20, 4=T30), second number (1–8) will be sampling location. For example, 30.06 will be a sample collected during the 30-day study from T0 at location 6. (ZIP 35 kb)

Additional file 13: OTU table sorted by location. This file contains OTU counts of all the families observed throughout the study for PMA treated (viable) and untreated (total) samples for various locations (bedroom, kitchen, bathroom and lab). (CSV 2 kb)

Additional file 14: OTU table sorted by location. This file contains OTU counts of all the families observed throughout the study for PMA treated (viable) and untreated (total) samples for various time points (T0, T13, T20 and T30). (TXT 132 kb)
