# Supplementary figures and images for: Human presence impacts fungal diversity of inflated lunar/Mars analog habitat
Source: Microbiome. 2017 Jul 11;5:62. doi: 10.1186/s40168-017-0280-8 (PMC5504618; doi:10.1186/s40168-017-0280-8)

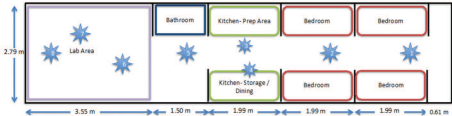

Supplement: Supplementary file 1 — Schematic representation of the ILMAH architecture with dimensions. The sampling locations are indicated with stars and numbers (1, 2: Bedroom; 3, 4: Kitchen; 5: Bathroom; and 6, 7, 8: Laboratory). (PDF 911 kb) [file 40168_2017_280_MOESM1_ESM.pdf]

Stress = 0.10

Day 0

Day 13

Day 20

Day 30

Day 0P

Day 13P

Day 20P

Day 30P

all

NMDS2

NMDS1

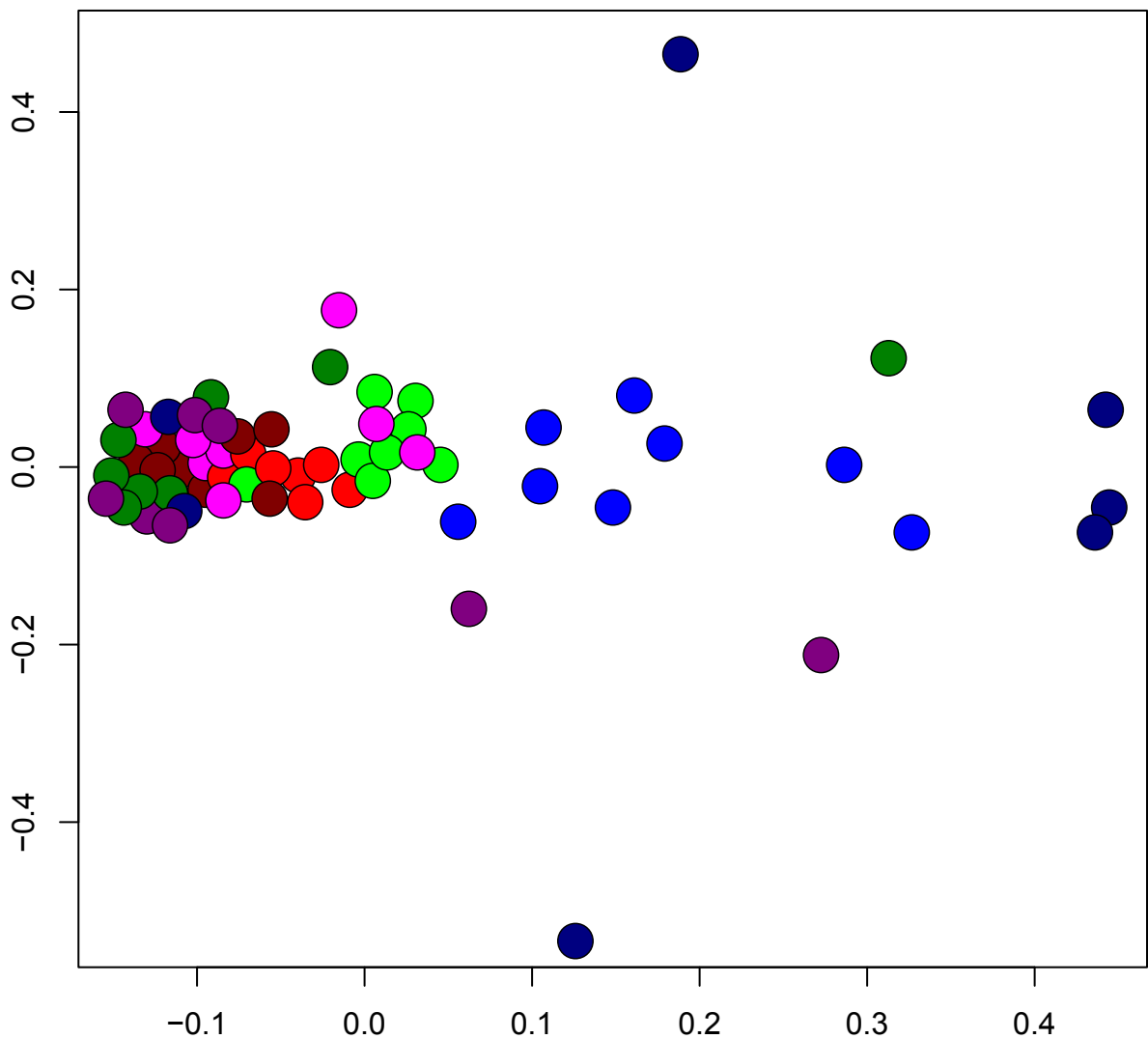

Supplement: Supplementary file 6 — NMDS ordinations based on Bray-Curtis distances between non-PMA- and PMA-treated samples taken at different time points. The analysis shows a significant difference between the PMA treated and not treated samples and between the different time points but not between the different locations. A “P” after the respective variable indicates that these are the samples treated with PMA. (PDF 44 kb) [file 40168_2017_280_MOESM6_ESM.pdf]

Stress = 0.049

● Day 0P

● Day 13P

● Day 30P

NMDS2

0.2

0.0

-0.2

0.0

0.2

0.4

0.6

NMDS1

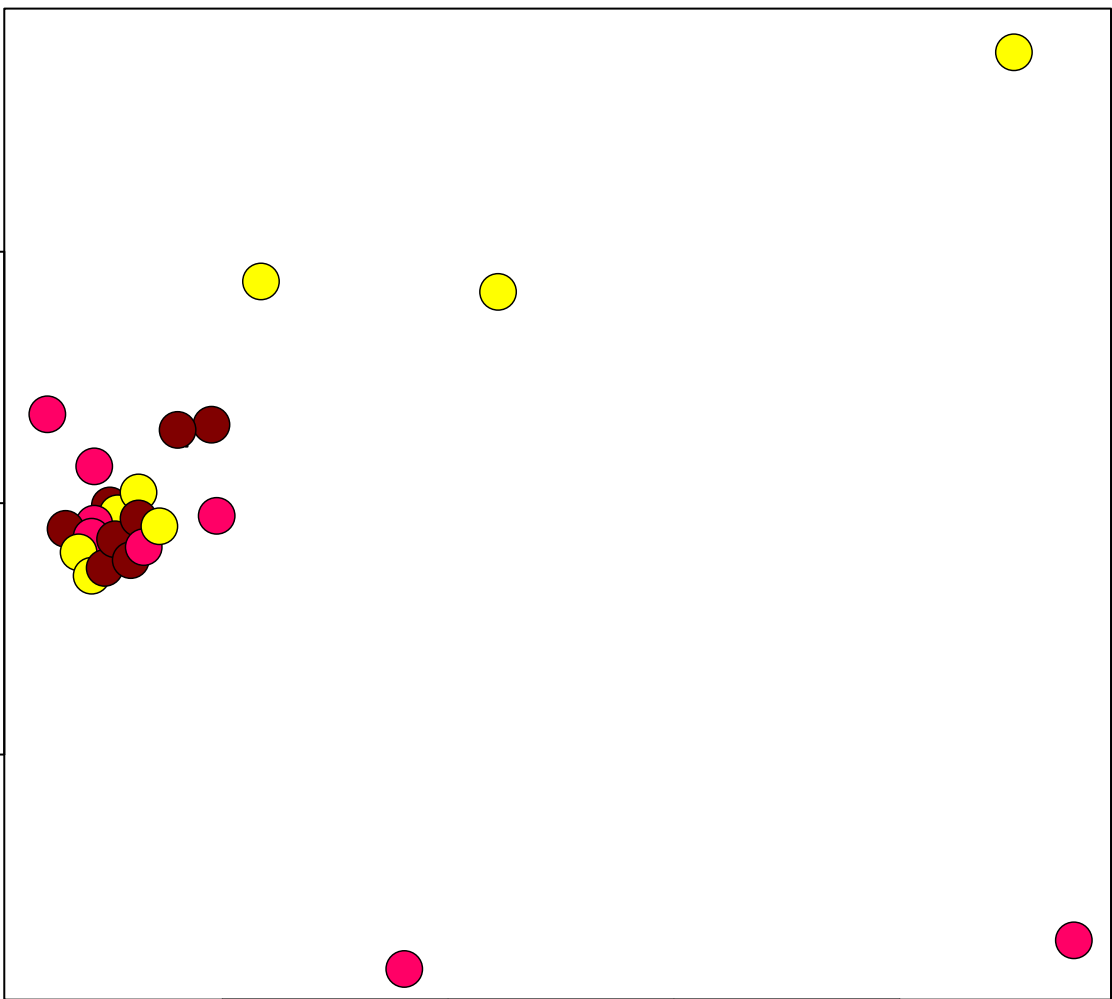

Supplement: Supplementary file 7 — NMDS ordinations based on Bray-Curtis distances between PMA treated samples without T20 taken at different time points. A “P” after the respective variable indicates that these are the samples treated with PMA. (PDF 30 kb) [file 40168_2017_280_MOESM7_ESM.pdf]
