# Supplementary material for: Human presence impacts fungal diversity of inflated lunar/Mars analog habitat
Source: Microbiome. 2017 Jul 11;5:62. doi: 10.1186/s40168-017-0280-8 (PMC5504618; doi:10.1186/s40168-017-0280-8)
Supplement: Supplementary file 4 — Cultivable fungal characteristics of ILMAH surface samples. Tables represent: a) CFU counts for each sampling area during consecutive sampling events (before crew occupation, Day 13, Day 20 and Day 30). Sampling areas are numbered 1-8. 1 and 2 correspond to bedroom area, 3, 4 – kitchen, 5 – bathroom and 6-8 lab. CFU counts are reported per meter square; b) CFU counts for specific ILMAH compartments. Table contains average CFU counts for each compartment: bedroom, kitchen, bathroom and lab area (for example CFU counts from area 1 and 2 at Day 13 were used to calculate the average CFU count for the bedroom compartment at Day 13). CFU counts are reported per meter square. (PDF 52 kb) [file 40168_2017_280_MOESM4_ESM.pdf]

Supplementary Table ST1. Cultivable fungal characteristics of ILMAH surface samples

a) CFU counts for each sampling area during consecutive sampling events

Sampling area	Cultivable fungal population (CFU/m^2)			
	T_0 (before crew occupation)	T_{13} (day 13)	T_{20} (day 20)	T_{30} (day 30)
1	1.58E+03	9.50E+02	BDL	BDL
2	1.30E+03	3.70E+02	4.10E+02	6.30E+02
3	7.78E+03	2.00E+02	5.55E+02	7.20E+02
4	2.89E+03	2.10E+03	8.75E+02	8.60E+02
5	3.29E+03	1.75E+03	5.70E+02	5.85E+02
6	9.39E+03	5.70E+03	7.20E+03	8.20E+02
7	8.77E+03	3.14E+03	1.26E+03	2.16E+03
8	2.52E+03	1.47E+04	1.48E+03	1.37E+03

b) CFU counts for specific ILMAH compartments

Time point	Location			
	Bedroom	Kitchen	Toilet	Lab area
T_0	1.44E+03	5.33E+03	3.29E+03	6.89E+03
T_{13}	6.60E+02	1.15E+03	1.75E+03	7.85E+03
T_{20}	2.05E+02	7.15E+02	5.70E+02	3.31E+03
T_{30}	3.15E+02	7.90E+02	5.85E+02	1.45E+03
