# Supplementary material for: Human presence impacts fungal diversity of inflated lunar/Mars analog habitat
Source: Microbiome. 2017 Jul 11;5:62. doi: 10.1186/s40168-017-0280-8 (PMC5504618; doi:10.1186/s40168-017-0280-8)
Supplement: Supplementary file 5 — Statistical analysis to compare cultivable fungal populations of the different (a) time points and (b) locations. (a) CFU counts of cultivable fungal populations observed at various time point were compared to each other to asses if there are any statistically significant changes in CFU counts over the course of time; (b) CFU counts of cultivable fungal populations observed at different compartments were compared to each other to asses if there are any statistically significant changes in CFU counts between locations. (PDF 43 kb) [file 40168_2017_280_MOESM5_ESM.pdf]

Supplementary Table ST2: Statistical analysis to compare fungal populations of the different a) time points and b) locations

a)

	T_0	T_{13}	T_{20}
T_{13}	0.0995		
T_{20}	0.008	0.1213	
T_{30}	0.0125	0.1391	0.4681

b)

	Bedroom	Kitchen	Lab
Kitchen	0.1506		
Lab	0.0021	0.0707	
Bathroom	0.1547	0.4653	0.1396
