# Supplementary material for: Human presence impacts fungal diversity of inflated lunar/Mars analog habitat
Source: Microbiome. 2017 Jul 11;5:62. doi: 10.1186/s40168-017-0280-8 (PMC5504618; doi:10.1186/s40168-017-0280-8)
Supplement: Supplementary file 8 — Statistical analysis of viable (PMA treated) samples to compare fungal populations of the different a) time points and b) locations. (a) Community profiles of viable fungal populations observed at various time point were compared to each other to asses if there are any statistically significant changes over the course of time; (b) Community profiles of viable fungal populations observed at different compartments were compared to each other to asses if there are any statistically significant changes between locations. Results marked with * are statistically significant. (PDF 44 kb) [file 40168_2017_280_MOESM8_ESM.pdf]

Supplementary Table ST3: Statistical analysis of viable (PMA treated) samples to compare fungal populations of the different a) time points and b) locations

a)

	T_0	T_{13}	T_{20}
T_{13}	0.199		
T_{20}	0.005**	0.031*	
T_{30}	0.059	0.443	0.044*

b)

	Bedroom	Kitchen	Lab
Kitchen	0.812		
Lab	0.642	0.804	
Bathroom	0.753	0.488	
