# Supplementary material for: Human presence impacts fungal diversity of inflated lunar/Mars analog habitat
Source: Microbiome. 2017 Jul 11;5:62. doi: 10.1186/s40168-017-0280-8 (PMC5504618; doi:10.1186/s40168-017-0280-8)
Supplement: Supplementary file 9 — Percent change of OTU counts at family level. Information about the percent changes of the OTU counts of selected families during consecutive time points is presented. (PDF 45 kb) [file 40168_2017_280_MOESM9_ESM.pdf]

Fungal Phylum	Fungal Family	Percent change (%)			
		T_0	T_{13}	T_{20}	T_{30}
Ascomycota	*Arthoniales*	0.27	0.00	0.00	0.00
	Capnodiales	0.00	0.04	0.00	0.50
	Chaetomiaceae	0.00	0.39	0.00	3.81
	Cucurbitariaceae	0.06	0.27	0.00	0.15
	Davidiellaceae	0.36	2.19	22.05	4.45
	Dothioraceae	0.03	0.10	11.44	0.08
	Hypocreaceae	0.02	0.07	0.00	1.26
	Hypocreales	0.02	0.14	0.00	0.65
	Leptosphaeriaceae	0.01	0.08	0.00	0.00
	Montanulaceae	0.06	0.22	0.00	0.01
	Nectriaceae	0.03	0.46	0.62	0.01
	Phaeosphaeriaceae	0.06	0.04	0.00	3.54
	Pleosporaceae	96.49	90.46	47.81	71.56
	Pleosporales	0.78	1.61	0.01	1.02
	Saccharomycetales	0.01	0.00	8.06	0.24
	Teratosphaeriaceae	0.01	0.00	0.00	5.17
	Trichocomaceae	0.01	1.24	8.12	0.06
	Trichosphaeriales	0.00	0.63	0.00	0.00
	Tubeufiaceae	0.00	0.27	0.00	0.00
	Unclassified	0.17	0.23	0.00	0.73
Basidiomycota	*Corticiaceae*	0.00	0.07	0.00	0.00
	Filobasidiaceae	0.11	0.00	1.49	0.51
	Peniophoraceae	0.00	0.00	0.00	1.26
	Sporidiobolales	0.31	0.11	0.00	2.81
	Tremellales	1.05	0.37	0.01	2.13
	Unclassified	0.07	0.78	0.39	0.03
